# Eukaryotic elongation factor-2 kinase expression is an independent prognostic factor in colorectal cancer

**DOI:** 10.1186/s12885-019-5873-0

**Published:** 2019-07-02

**Authors:** Tung H. Ng, Kathy W. Y. Sham, Chuan M. Xie, Simon S. M. Ng, Ka F. To, Joanna H. M. Tong, Wing Y. Z. Liu, Lin Zhang, Matthew T. V. Chan, William K. K. Wu, Christopher H. K. Cheng

**Affiliations:** 1 School of Biomedical Sciences, The Chinese University of Hong Kong, Hong Kong, China; 2 Institute of Hepatobiliary Surgery, Southwest Hospital, Third Military Medical University (Army Medical University), Chongqing, China; 3 State Key Laboratory of Digestive Diseases, Centre for Gut Microbiota Research, Institute of Digestive Diseases and LKS Institute of Health Sciences, The Chinese University of Hong Kong, Hong Kong, China; 4 Department of Surgery, The Chinese University of Hong Kong, Hong Kong, China; 5 Department of Anatomical & Cellular Pathology, The Chinese University of Hong Kong, Hong Kong, China; 6 Department of Anaesthesia and Intensive Care, The Chinese University of Hong Kong, Hong Kong, China; 7 CUHK Shenzhen Research Institute, Shenzhen, China

**Keywords:** EEF2K, Colon cancer, Survival, TNM staging, Biomarker

## Abstract

**Background:**

Prognostication of patients with colorectal cancer (CRC) currently relies on tumor-node-metastasis (TNM) staging but clinical outcomes of patients of the same histoclinical stage are heterogeneous. It is therefore imperative to devise novel molecular tests to stratify CRC patients. Our previous work demonstrated that eukaryotic elongation factor-2 kinase (EEF2K) is a tumor suppressor in CRC. Herein, we investigated EEF2K expression in CRC and determined its relationship with clinicopathological parameters.

**Methods:**

Quantitative RT-PCR and Westerns blots were used to examine EEF2K expression in primary tumor and the adjacent non-tumor tissues of CRC patients (*n* = 20). Kaplan-Meier curves and Cox regression analysis were used to assess the association between clinical outcomes of CRC patients and EEF2K protein expression determined by immunohistochemistry on tissue microarray (*n* = 151).

**Results:**

EEF2K was significantly downregulated at both mRNA and protein levels in tumors of CRC patients. Univariate Cox regression analysis revealed that CRC patients with high tumor grade, advanced TNM staging and low EEF2K expression were associated with worse overall survival. Multivariate analysis further demonstrated that low EEF2K expression was an independent factor for predicting poorer overall survival in CRC patients (*p* = 0.014; Hazard ratio = 2.951; 95% confidence interval: 1.240–7.024). The 5-year survival rate was 82.8% in the EEF2K-high-expression group versus 63.9% in the EEF2K-low-expression group (*p* = 0.0118). The association of overall survival with EEF2K expression in CRC patients was verified in The Cancer Genome Atlas (TCGA) cohort.

**Conclusions:**

EEF2K is downregulated in CRC and its expression can be employed as a prognostic marker for CRC patients independent of TNM staging.

**Electronic supplementary material:**

The online version of this article (10.1186/s12885-019-5873-0) contains supplementary material, which is available to authorized users.

## Background

Colorectal cancer (CRC) is the second most common malignant disease and the fourth leading cause of cancer-related death worldwide, accounting for more than 650,000 deaths annually [1]. In particular, CRC incidence in many Asian countries has increased 2- to 4-fold over the last two decades [2]. Despite the recent advancements in CRC treatment, up to 50% patients whom underwent tumor resection experience cancer recurrence, among which another half subsequently developed virtually incurable metastasis. The prognostication of CRC patients remains a clinical challenge with tumor-node-metastasis (TNM) staging as the most commonly used prognostic tool in the clinical setting. However, the clinical value of this system in guiding suitable therapy has recently been questioned. For instance, adjuvant therapy is recommended for all stage III patients with CRC but it remains controversial for stage II patients as its toxicities may outweigh benefits. It is therefore pivotal to identify early-stage CRC patients with predicted gloomy outcomes pending for more aggressive treatment. In this respect, the utility of molecular predictive and prognostic markers has helped forecast clinical outcome and treatment responsiveness for deciding better interventions for CRC patients. For example, *KRAS* mutation has been established as a negative predictor for response to epidermal growth factor receptor-targeted therapy in patients with metastatic CRC [3]. Also, programmed death (PD)-ligand 1 (L1) expression on tumor cells predicts efficacy of PD-L1 and PD-1 inhibition-based therapies in different types of cancer [4]. Thus, the development of novel prognostic marker for clinical outcome prediction in CRC is highly warranted.

Eukaryotic elongation factor-2 kinase (EEF2K) is a calcium/calmodulin-dependent protein kinase that plays a role in regulating protein synthesis [5]. EEF2K phosphorylates its downstream target eukaryotic elongation factor-2 (EEF2), a GTPase that promotes the translocation of the nascent protein chain from A site to P site on ribosome in elongation phase during mRNA translation [6]. The phosphorylation of EEF2 at threonine 56 by EEF2K inhibits the interaction of EEF2 with ribosome and thereby inhibiting protein elongation. In our recent study, a tumor-suppressive role of EEF2K was observed in CRC, where silencing of EEF2K induced a pro-survival autophagic response through the AMPK-ULK pathway and promoted CRC growth through increasing cell size, viability and clonogenicity. In contrast, overexpressing EEF2K reduced colon cancer cell viability and potentiated the anti-tumor efficacy of the chemotherapeutic drug oxaliplatin [7]. Consistently, De Gassart and co-workers reported that nelfinavir exerted its anti-tumor effect in colon cancer cells in an EEF2K-dependent manner [8]. An in vivo study by Faller and his colleagues also revealed that EEF2K activation led to growth arrest in APC-deficient colorectal adenomas [9]. All these pieces of evidence support EEF2K as a tumor-suppressor gene in CRC. However, the clinical significance of EEF2K downregulation in CRC remains to be established.

In the current study, we investigated the expression of EEF2K in CRC and delineated its relationship with clinicopathological parameters, including survival, in patients with CRC.

## Methods

### Clinical samples

Twenty pairs of primary tumor tissues and matched adjacent non-tumor tissues were collected during operation from CRC patients who were admitted to the Prince of Wales Hospital, Shatin, Hong Kong. All specimens were immediately frozen in liquid nitrogen and stored at -80 °C until use. For tissue microarrays, formalin-fixed, paraffin-embedded archived CRC tissues were used. Use of these tissues had been approved by the Joint Chinese University of Hong Kong—New Territories East Cluster Clinical Research Ethics Committee. Informed written consents from patients were obtained.

### The Cancer Genome Atlas (TCGA) cohort

Expression data and clinical information were collected from the TCGA open access data directory. Reads mapped to EEF2K (level 3 data) were used to quantify EEF2K mRNA expression levels and normalized by transcripts per million mapped reads (TPM).

### RNA extraction, reverse transcription and real-time PCR

Total RNA was extracted from aforementioned matched samples using TRIzol reagent (Life Technologies) following manufacturer’s protocol. First-strand complementary DNA was synthesized from the extracted RNA using PrimeScript™ RT Reagent kit (TaKaRa) with a protocol suggested by the manufacturer. EEF2K levels were quantified using Power SYBR® Green PCR master mix (Applied Biosciences) on ABI Quantstudio™ 7 Flex Real Time PCR System (Thermo-cycling condition: 95 °C for 10 min, followed by 40 cycles of 95 °C for 15 s and 60 °C for 1 min). Relative expression was calculated using 2^-∆∆Ct^ method using β-actin for normalization.

### Protein extraction and Western blots

Total protein was extracted by homogenizing the tissues in radioimmunoprecipitation assay buffer (150 mM sodium chloride, 50 mM Tris, 1% Triton-100, 0.5% sodium deoxycholate, 0.1% sodium dodecyl sulphate, pH 7.6) containing PhosSTOP™ phosphatase inhibitors (Roche) and cocktail protease inhibitors (Sigma). After centrifugation at 12,000⨯*g* for 20 min, the supernatant was mixed and heated with Laemmli buffer. Proteins were resolved by sodium dodecyl sulphate-polyacrylamide gel electrophoresis and transferred onto a nitrocellulose membrane (Pall Corporation), which was then incubated with antibodies against EEF2K (Cell Signaling Technology) or β-actin (Santa Cruz Biotechnology) at 4 °C overnight. The membranes were then washed and incubated with horseradish peroxidase-conjugated secondary antibodies (Cell Signaling Technology) and the bands were visualized with enhanced chemiluminescent reagent (Advansta).

### Immunohistochemistry for EEF2K

Tissue microarrays comprising formalin-fixed paraffin-embedded tumor tissue sections from 160 cases of CRC were subjected to immunohistochemistry for determining EEF2K protein levels using Histostain® Plus LAB-SA Detection System (ThermoFisher) with a slightly modified protocol. Briefly, tissue sections were deparaffinized and rehydrated, and incubated in 3% hydrogen peroxide for 10 min to block endogenous peroxidase activity. Antigens were retrieved by microwave in sodium citrate buffer (10 mM sodium citrate, 0.05% Tween 20, pH 6.0) for 5 min. After cooling to room temperature, sections were incubated in 10% goat serum (Dako) for 2 h and then with antibody against EEF2K (Novartis) at 4 °C overnight, and substantially washed with phosphate-buffered saline with 0.025% Tween 20. Then, sections were incubated with biotinylated secondary antibodies, followed by streptavidin-conjugated horseradish peroxidase. After washing, signals were developed using 3, 3′-diaminobenzidine as the chromogen and counterstained with hematoxylin. Expression levels of EEF2K on each specimen were evaluated and scored according to the percentage of epithelial cells possessing positive signals and the intensity of the positive signals. Percentage of positive signal was graded as: 1 (5–10%); 2 (11–20%); 3 (> 20%). Intensity of signal was graded as: 0 (none); 1 (weak); 2 (moderate); 3 (strong). EEF2K score was calculated as the product of the two grades. Sections with score ≥ 2 were classified as EEF2K-positive and < 2 as EEF2K-negative.

### Statistical analysis

Chi-square test was used to determine the correlation between grouped EEF2K expression with categorical clinicopathological parameters. Student’s *t* test and one-way analysis of variance was used to compare means of continuous data. Kaplan-Meier analysis and log-rank test were used to evaluate the correlation of EEF2K expression with patients’ survival. Univariate and multivariate Cox regression analyses were used to assess the prognostic value of EEF2K expression and other clinicopathological parameters in predicting survival outcome. The proportional hazard assumption of the Cox analysis was verified by assessing the scaled Schoenfeld residuals and time-dependent covariates for each predictor. Survival-related statistical analyses were conducted using SPSS 22.0 software while other statistical tests were conducted with GraphPad Prism 6. Results were considered significant when two-sided *p* value < 0.05. Experiments were conducted for at least 3 times.

## Results

### EEF2K was downregulated in CRC at mRNA and protein levels

**Fig. 1:**
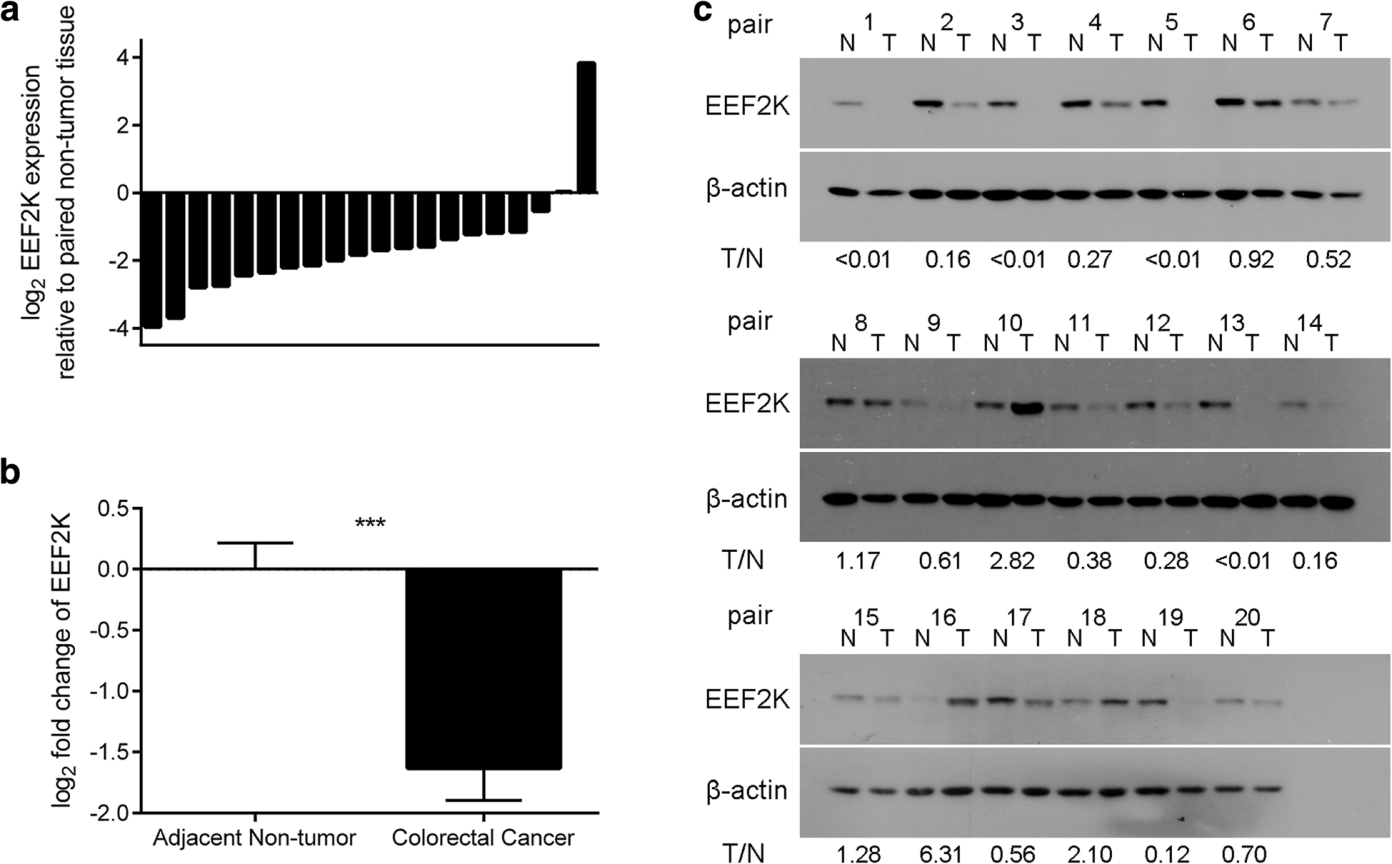
Downregulation of EEF2K in human CRC. **a** Individual and **b** overall *EEF2K* mRNA expression in 20 pairs of CRC tissue and paired non-tumor tissue evaluated by real-time PCR analysis. Negative value denotes downregulation in tumor tissue over normal counterparts. **c** Western blots of EEF2K protein in 20 pairs of matched adjacent non-tumor (N) and tumor (T) tissues. Quantification was performed using ImageJ software and expressed as ratio of T over N for each pair of tissue. ***, *p* < 0.001 against adjacent non-tumor tissue by two-sided paired *t*-test

To unravel the potential dysregulation of EEF2K in CRC tissues, the relative mRNA and protein expression levels of EEF2K were assessed by real-time PCR and Western blots, respectively. Eighty-five percent of the cases (17 out of 20) showed a more than two-fold downregulation of *EEF2K* mRNA in cancerous tissues as compared to the adjacent non-tumor tissues (Fig. 1a). In general, the downregulation of *EEF2K* in tumor tissues was significant (Fig. 1b). At protein level, consistent with the mRNA analysis, EEF2K was significantly reduced in 75% of the cases (15 out of 20) in primary tumor tissue relative to the paired non-tumor tissues (Fig. 1c). The downregulation of EEF2K in CRC tissues was not associated with patients’ age, gender, tumor location, tumor grade, tumor stage or nodal stage (Table 1).

**Table 1:** Change in EEF2K protein expression pattern between paired tissues and correlation with patients’ clinicopathological factors

Variable	Upregulation in tumor *N* (%)	Downregulation in tumor *N* (%)	*p*
Age at operation (Mean ± SD)	72.0 ± 10.3	68.2 ± 8.4	0.417
Gender			
Male	3 (25.0)	9 (75.0)	1.000
Female	2 (25.0)	6 (75.0)	
Tumor location			
Right colon	3 (42.9)	4 (57.1)	0.240
Left colon	2 (25.0)	6 (75.0)	
Rectum	0 (0)	5 (100)	
Tumor grade			
Poor or mucinous	1 (50.0)	1 (50.0)	0.447
Moderate	4 (22.2)	14 (77.8)	
Tumor stage			
T1 or T2	1 (50.0)	1 (50.0)	0.447
T3 or T4	4 (22.2)	14 (77.8)	
Nodal stage			
N0	2 (20.0)	8 (80.0)	0.864
N1	2 (28.6)	5 (71.4)	
N2	1 (33.3)	2 (66.7)	

*N* number, *SD* standard deviation

### Immunohistochemical staining assessment and correlation analysis of EEF2K expression with patients’ clinicopathological factors

**Fig. 2:**
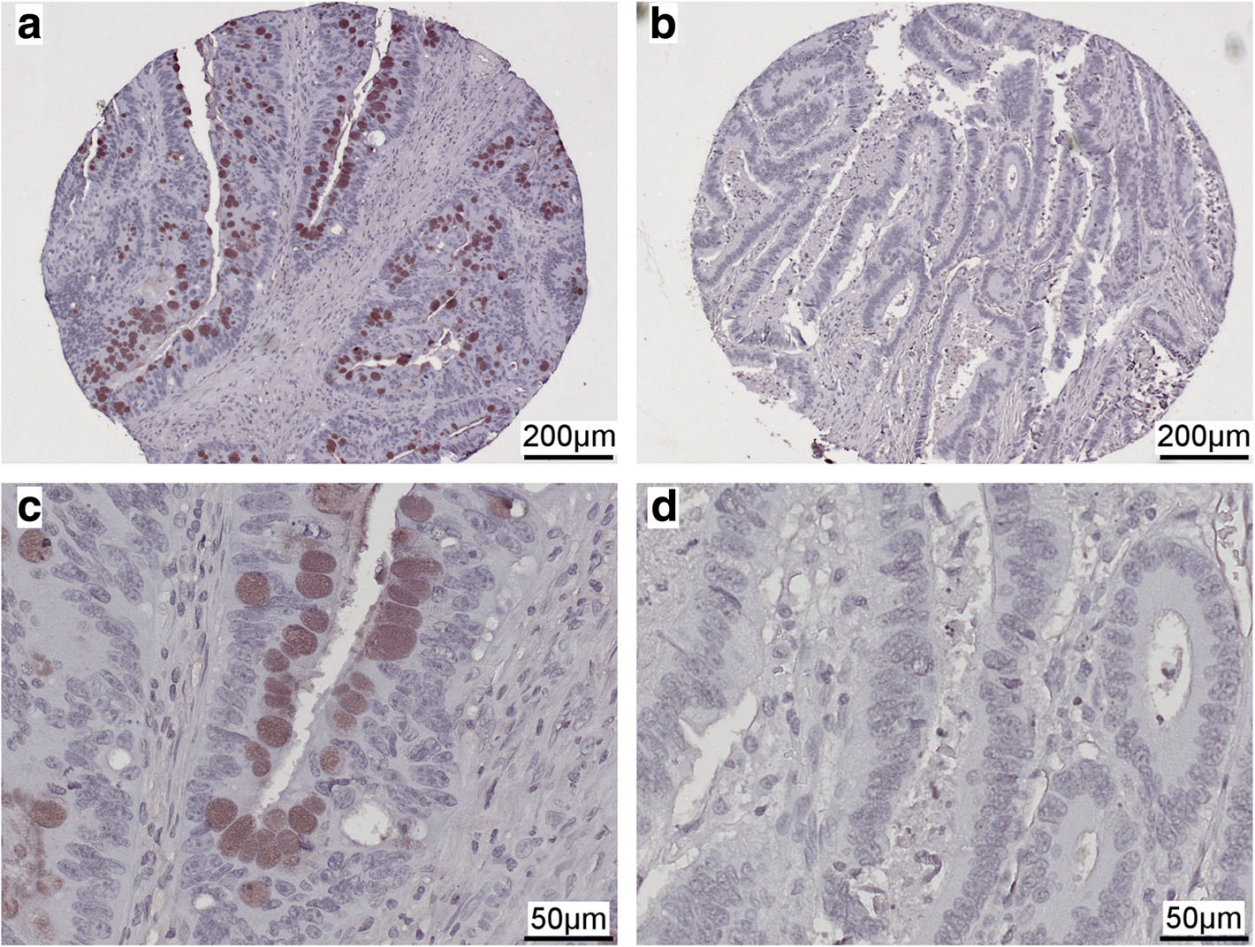
Representative micrographs of immunohistochemical staining of EEF2K protein in CRC tissues. **a** Positive and **b** negative EEF2K expression (magnification, m = 100⨯). **c** and **d** are higher magnification of **a** and **b**, respectively (m = 400⨯ of original sections)

Tissue microarrays comprising 160 CRC tissues were subjected to immunohistochemistry for detecting EEF2K protein expression. In addition to tissues lost during staining and samples with incomplete information, patients who survived less than 3 months after surgery were filtered out to exclude death caused by surgical complications. Finally, 151 cases were enrolled for the current analysis, of which 19.2% cases (29 out of 151) were classified as EEF2K-positive and 80.8% cases (122 out of 151) as EEF2K-negative based on percentage and intensity of positive staining (Fig. 2). EEF2K expression was not correlated with patients’ age, gender, tumor location, tumor grade, TNM staging or microsatellite instability (MSI) status (Table 2).

**Table 2:** EEF2K expression and correlation with patients’ clinicopathological factors in our cohort

Variable	Positive expression *N* (%)	Negative expression *N* (%)	*p*
Age at operation (Mean ± SD)	68.2 ± 9.7	67.4 ± 12.3	0.766
Gender			
Male	18 (20.7)	69 (79.3)	0.589
Female	11 (17.2)	53 (82.8)	
Tumor location			
Right colon	11 (25.6)	32 (74.4)	0.383
Left colon	3 (12.5)	21 (87.5)	
Rectum	15 (17.9)	69 (82.1)	
Tumor grade			
Poor	0 (0)	5 (100)	0.584
Moderate and well	29 (19.9)	117 (80.1)	
Tumor stage			
T1	2 (50)	2 (50)	0.247
T2	5 (29.4)	12 (70.6)	
T3	17 (16.3)	87 (83.7	
T4	5 (19.2)	21 (80.8)	
Nodal stage			
N0	21 (24.7)	64 (75.3)	0.143
N1	5 (11.1)	40 (88.9)	
N2	3 (14.3)	18 (85.7)	
Metastasis			
No	27 (19.9)	109 (80.1)	0.737
Yes	2 (13.3)	13 (86.7)	
Microsatellite instability			
Microsatellite stable	23 (18.9)	99 (81.1)	0.690
MSI-Low	2 (22.2)	7 (77.8)	
MSI-High	1 (9.1)	10 (90.9)	

*N* number, *SD* standard deviation, *MSI* microsatellite instability

### Low EEF2K expression was correlated with worse clinical outcome

**Fig. 3:**
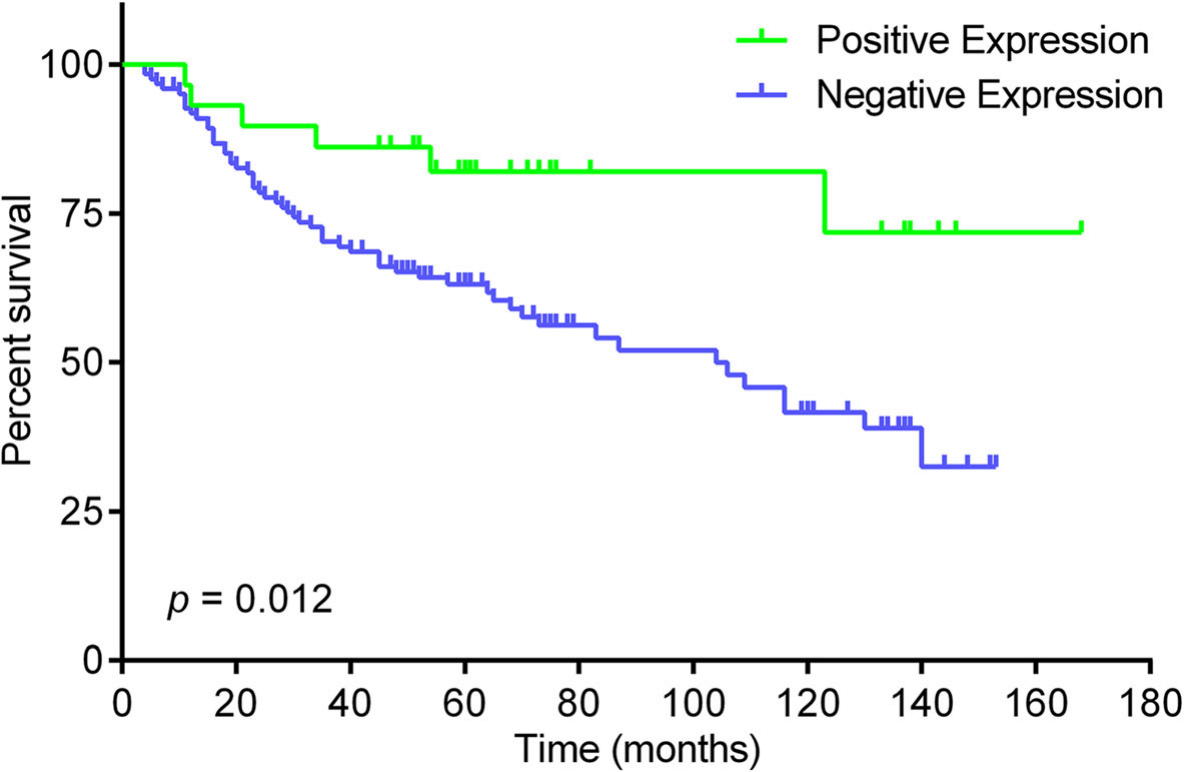
Kaplan-Meier curves for overall survival in relation to EEF2K expression in our cohort. *P* value was obtained by log-rank test (*n* = 151). Each tick mark represents a censored case

To evaluate the clinical relevance of EEF2K expression in CRC, we examined the correlation of EEF2K expression with clinical outcomes of the patients in our cohort. Kaplan-Meier analysis showed that patients in the EEF2K-negative group exhibited significantly worse overall survival than those in the EEF2K-positive group (Fig. 3). We then assessed the prognostic potential of EEF2K expression in predicting overall survival with Cox regression analysis. Univariate analysis of our patient cohort indicated that high tumor grade, advanced TNM staging and low EEF2K expression were predictors for shortened overall survival. In multivariate analysis, low EEF2K expression was shown to be an independent predictor for poorer overall survival (Hazard ratio = 2.951, with reference to EEF2K positive group; 95% confidence interval: 1.240–7.024; *p* = 0.014) after controlling tumor grade, TNM staging together with age and gender of the patients for potential confounding effects (Table 3).

**Table 3:** Cox regression analysis for predictors of overall survival in patients with CRC in our cohort

Variable	Univariate			Multivariate		
	HR	95% CI	*p* value	HR	95% CI	*p* value
Gender (Female vs Male)	1.11	0.69–1.82	0.673	0.87	0.67–1.13	0.289
Age (≥65 vs < 65)	1.01	0.61–1.67	0.963	1.16	0.67–2.00	0.606
Tumor location (colon vs rectum)	1.09	0.64–1.84	0.751			
Tumor grade (poor vs moderate to well)	18.8	6.99–50.7	< 0.001*	3.27	0.98–10.9	0.053
Tumor stage (T4 vs T1 to T3)	4.78	2.77–8.27	< 0.001*	3.81	1.92–7.56	< 0.001*
Lymph node invasion (Presence vs Absence)	3.11	1.87–5.17	< 0.001*	2.54	1.47–4.37	0.001*
Metastasis (Presence vs Absence)	9.95	5.25–18.9	< 0.001*	4.04	1.70–9.61	0.002*
MSI (MS unstable vs MS stable)	0.53	0.22–1.23	0.138			
EEF2K expression (Negative vs Positive)	2.81	1.21–6.51	0.016*	2.95	1.24–7.02	0.014*

*HR* hazard ratio, *CI* confidence interval, *MS* microsatellite, *MSI* microsatellite instability, * *p* < 0.05

### Validating the expression and prognostic significance of EEF2K in TCGA cohort

**Fig. 4:**
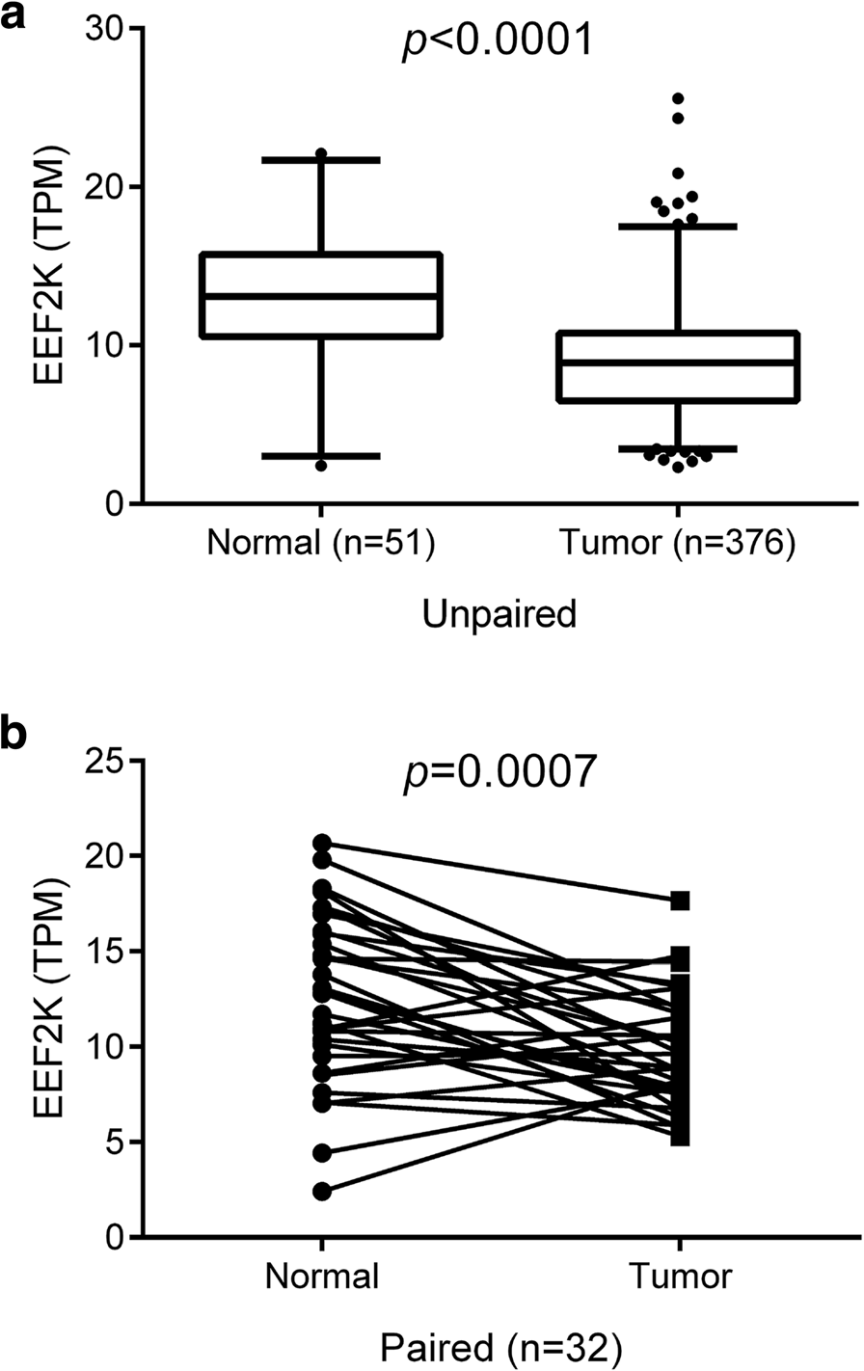
Downregulation of EEF2K in CRC in TCGA cohort. EEF2K expression between (**a**) unpaired and (**b**) paired normal and colorectal tumor tissues from TCGA RNA sequencing data. The whisker spans the 95% confidence interval. *P* values were obtained by two-sided unpaired and paired *t*-test accordingly

**Fig. 5:**
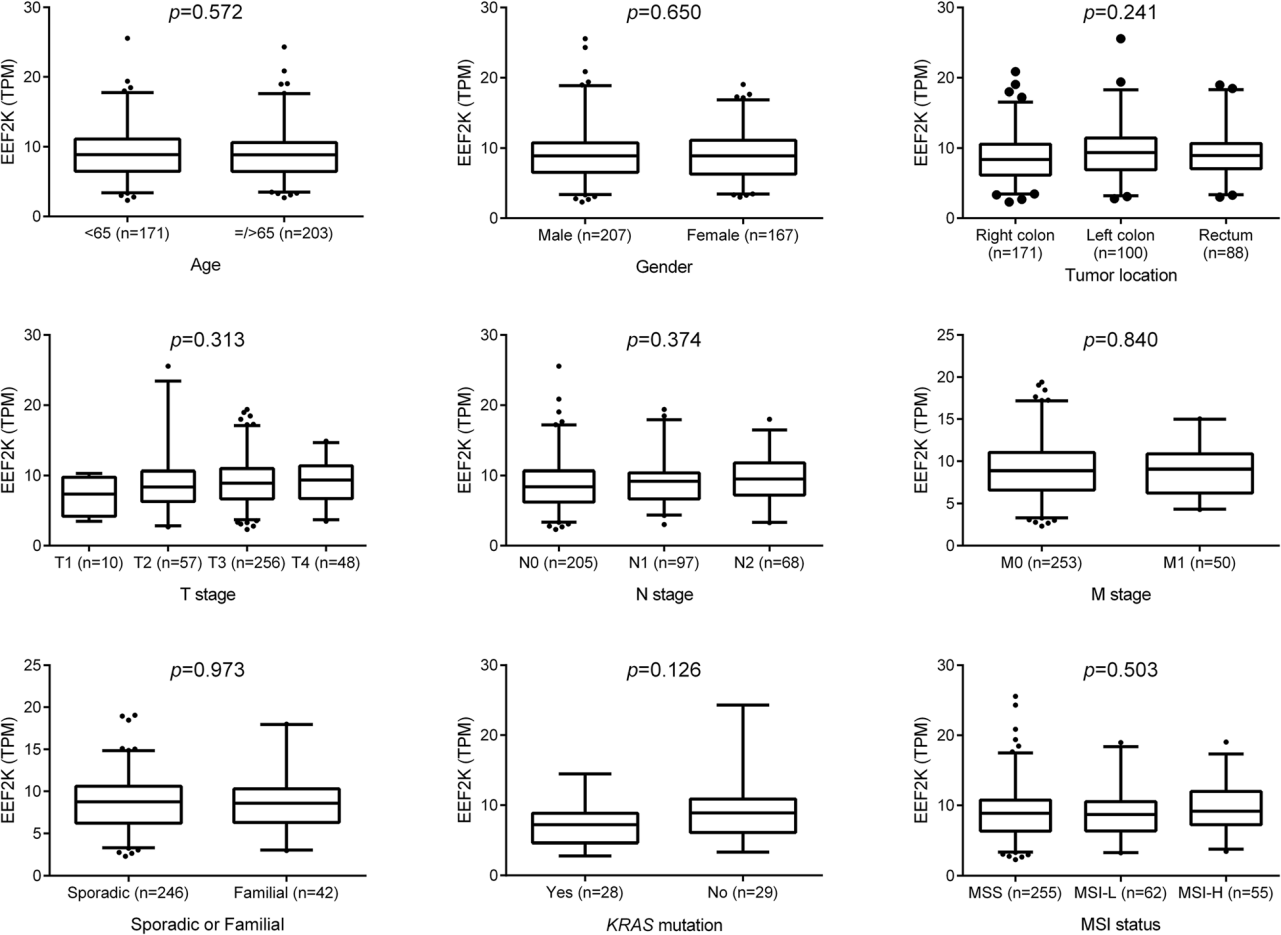
Association of EEF2K expression with patients’ clinicopathological factors and mutational status in TCGA cohort. EEF2K expression was obtained from TCGA RNA sequencing data and expressed as transcripts per million mapped reads (TPM). *P* values were obtained by student’s *t* test and one-way analysis of variance when comparing factors of two groups and more than two groups respectively. The whisker spans the 95% confidence interval

**Fig. 6:**
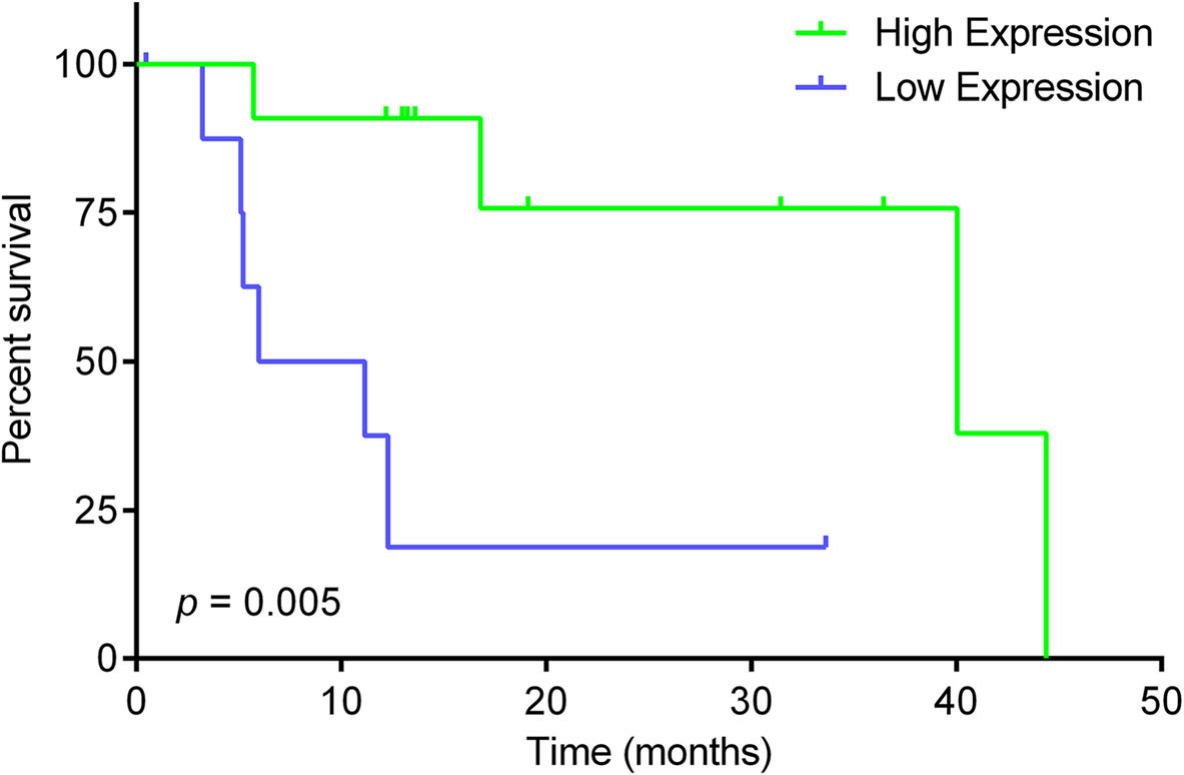
Kaplan-Meier curves for overall survival in relation to EEF2K expression in TCGA cohort. Analysis was performed on patients with Stage IV CRC. *P* value was obtained by log-rank test (*n* = 20). Each tick mark represents a censored case

The expression of EEF2K and its association with overall survival of CRC patients were further studied using The Cancer Genome Atlas (TCGA) cohort. Data of RNA sequencing, together with the clinical features and survival information of CRC patients, were obtained from the TCGA Data Portal. Our analysis demonstrated that EEF2K expression was significantly downregulated in CRC in both unpaired and paired comparison between normal and tumor tissues (Fig. 4). The downregulation of EEF2K was common but not associated with patients’ clinical characteristics (age, gender, tumor location, TNM staging), CRC subtypes (sporadic vs familial) or mutational status (MSI status and mutation of *KRAS*, *BRAF*, *APC* and *TP53*) (Fig. 5 and Additional file 1). The clinical relevance of EEF2K expression in CRC patients was then evaluated. Patients with the highest 20% *EEF2K* mRNA expression (high expression group) and the lowest 20% (low expression group) among the cohort were selected and their survival outcomes were compared (Additional file 2). Our analysis indicated that, after stratifying at stage IV CRC (AJCC), patients in the low expression group had significantly worse overall survival than those in the high expression group (20 cases; Fig. 6).

## Discussion

EEF2K, together with other enzymes involved in protein synthesis, were traditionally known as cellular housekeepers that play a vital role in cell viability [10]. In our study, we further extended the role of EEF2K as a prognostic biomarker for CRC in which low EEF2K expression foreshadowed worse overall survival independent of other clinicopathological parameters, including age, gender and TNM staging of the patients. This observation is consistent with our previous finding that EEF2K functioned as a tumor-suppressor gene in CRC by inhibiting autophagic survival and synergized with oxaliplatin (a commonly used chemotherapeutic drug in CRC management) to induce colon cancer cell apoptosis [7]. Our findings were further validated in TCGA cohort, in which EEF2K was also downregulated in CRC without association with patients’ clinical features, CRC subtypes or mutational status as in our cohort. However, its clinical relevance with survival outcome of CRC patients was only noted in patients with stage IV CRC. It is aware that the methodologies in assessing EEF2K expression level, and also the ethnicity of patients and underlying genetic background were different and may account for the discrepancy. Also, it should be noted that multiple comparisons were not adjusted for this analysis. Additional dedicated studies are needed for further confirmation. Despite all these, our findings indicated that EEF2K expression, alongside other molecular prognostic markers such as microsatellite instability [11], *BRAF* mutation status [12], gene expression profiling [13] and multi-gene mutation signature [14], might supplement TNM staging in the future for more accurate prognostication and patient stratification.

The dysregulation of EEF2K and its clinical relevance have been documented in other cancer types. It is noteworthy that, contrary to its tumor-suppressive role in CRC, EEF2K was frequently found to function as an oncogene in other cancer types. Ashour and colleagues reported an overexpression of EEF2K in pancreatic cancer [15]. Meric-Bernstam and colleagues found that EEF2K was overexpressed in breast cancer and, importantly, the expression of EEF2K was positively correlated with poor prognosis in breast cancer patients [16]. A similar finding was also reported in brain cancer by Leprivier and his colleagues [17]. The tissue-specific functions of EEF2K may provide a plausible explanation for such discrepancy. While EEF2K overexpression has been shown to promote cancer cell survival in face of nutrient starvation by inhibiting protein synthesis resulted in limitation of cellular energy exhaustion [18], mTOR complex 1-mediated inhibition of EEF2K was found to be essential for the proliferation of APC-deficient but not wild-type enterocytes [9]. This distinctive synthetic lethality circuit created by APC inactivation in CRC strongly supports the intestine-specific tumor-suppressive effect of EEF2K and its association with better prognosis in CRC patients. This finding also suggests that EEF2K may function as a tumor suppressor in other cancer types, such as endometrial [19] and gastric cancers [20], in which APC inactivation plays a significant oncogenic role. The prognostic role of EEF2K expression in these cancer types also warrants further investigation to support the notion.

## Conclusions

Taken together, we found that EEF2K downregulation is independently associated with worse overall survival in CRC patients. To the best of our knowledge, it is the first time demonstrating the clinical relevance of EEF2K expression in CRC patients. The use of EEF2K as a prognostic marker could be promising in identifying high-risk CRC patients to improve their survival with more aggressive treatment.

## Additional files

Additional file 1: Association of EEF2K expression with *BRAF*, *APC* and *TP53* gene mutation in TCGA cohort. EEF2K expression was obtained from TCGA RNA sequencing data. *P* values were obtained by student’s *t* test. The whisker spans the 95% confidence interval. (TIF 220 kb)

Additional file 2: Significance levels and different grouping cut-offs of EEF2K expression in survival analysis. Analysis was performed from data in TCGA cohort. Patients were grouped into high and low EEF2K expression groups by different cut-off values of EEF2K expression based on percentiles. n^th^ percentile indicates that patients fall into the lowest n% and highest n% in EEF2K expression were selected and grouped into low expression and high expression group respectively. Their overall survivals were compared and significance levels (or *p* values) were obtained by log-rank tests accordingly. **, *p* < 0.01. (TIF 90 kb)

## Data Availability

The datasets used and/or analysed during the current study are available from the corresponding author on reasonable request.

## Abbreviations

CRC: Colorectal cancer
EEF2: Eukaryotic elongation factor-2
EEF2K: Eukaryotic elongation factor-2 kinase
MSI: Microsatellite instability
RT-PCR: Reverse transcription-polymerase chain reaction
TNM: Tumor-node-metastasis
